# Statin Use in Heart Failure: A Rebirth?∗

**DOI:** 10.1016/j.jacadv.2023.100387

**Published:** 2023-06-30

**Authors:** R. Scott Wright

**Affiliations:** Division of Preventive Cardiology and the Department of Cardiology, Mayo Clinic, Rochester, Minnesota, USA

**Keywords:** heart failure, statins

**Figure:**
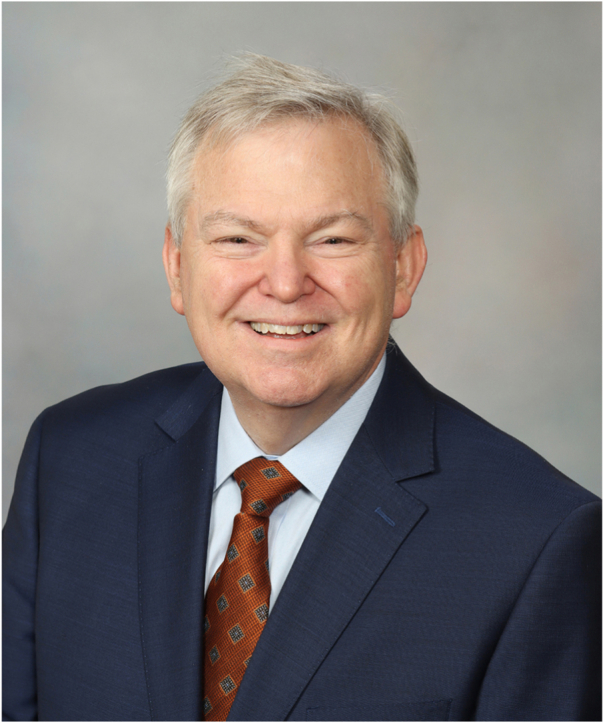

One of the puzzling challenges in clinical cardiology has been the difficulty with regard to the demonstration of a benefit from statin therapy in patients with heart failure, even in those with ischemic heart failure. Nearly 15 years ago, the Controlled Rosuvastatin Multinational Trial in Heart Failure (CORONA)^1^ and the Gruppo Italiano per lo Studio del Sopravvivenza nell’Insufficienza cardiaca (GISSI-HF)^2^ trials evaluated statin therapy in heart failure populations. CORONA largely randomized those with ischemic heart failure while GISSI-HF included ischemic and nonischemic heart failure patients. Both studies were deemed negative and heralded an end to an otherwise long run of successful clinical trials evaluating statin therapy in a variety of clinical conditions.

## GISSI-HF and CORONA

The GISSI-HF trial randomized 4,574 patients with ischemic and nonischemic heart failure to rosuvastatin 10 mg daily or placebo for 3.9 years. The primary end points were time to death and time to death or admission for heart failure. The hazard ratios (HRs) for the time to death was 1.00 (95% CI: 0.898-1.122, *P* = 0.943) and time to death or admission for heart failure was 1.01 (95% CI: 0.908-1.112, *P* = 0.903). The CORONA trial randomized 5,011 participants with ischemic heart failure to rosuvastatin 10 mg or placebo for a median time of 32.8 months with a primary outcome composite of cardiovascular death, nonfatal myocardial infarction, and/or nonfatal stroke. The primary end point HR was 0.92 (95% CI: 0.83-1.02, *P* = 0.12). While the primary end point was not significantly reduced, there were fewer hospitalizations in those randomized to rosuvastatin.

Multiple hypotheses have been advanced about why statin therapy failed to reduce significantly the measured primary end points in both trials but especially CORONA.^3^^,^^4^ Perhaps it was a type 2 statistical error due to an inadequate sample size or number of accrued events. Others have suggested that the recruited population was too old and/or that symptomatic advanced heart failure may produce clinical sequela which cannot be mitigated by statin therapy. Regardless, most clinicians remained perplexed as to why statin therapy was not demonstrated as beneficial in the heart failure population.

## Statin therapy revisited

Given the clinical conundrum of a lack of statin efficacy in heart failure juxtaposed against a body of robust evidence of benefit in atherosclerotic cardiovascular disease (ASCVD) patients, in this issue of *JACC: Advances*, Anderson et al^5^ at the Intermountain Health System evaluated the impact of statin therapy across a broad sample of heart failure patients using a real-world evidence (RWE) model based upon the Intermountain Medical Center extensive electronic health record covering this large clinical health care system.

**Figure 1:**
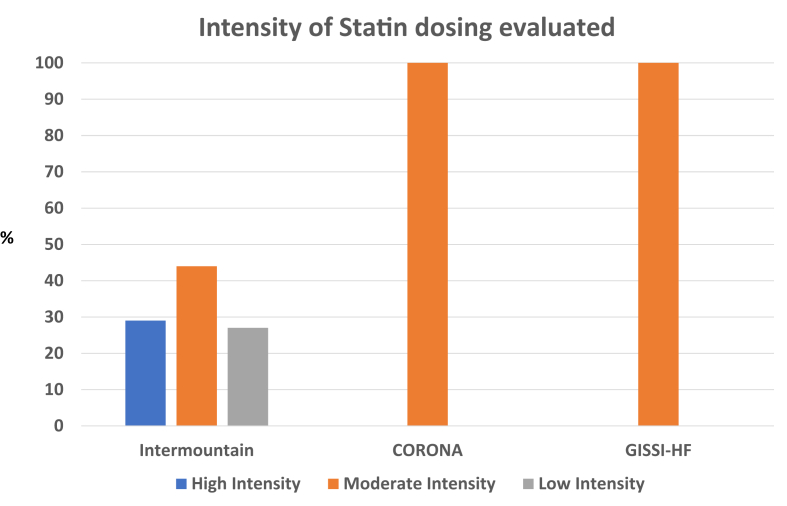
**Statin Doses Utilized in Intermountain Study and the CORONA and GISSI-HFTrials**

**Figure 2:**
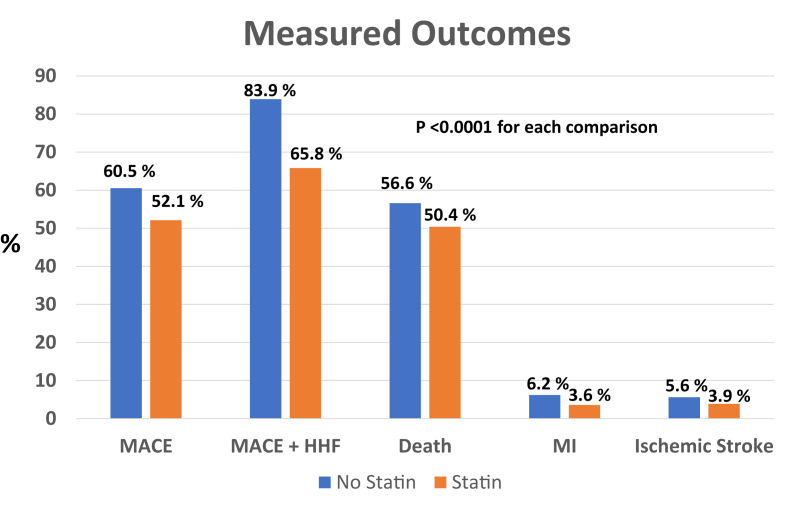
**Endpoints and Impact From Associated StatinUse**

They sought to determine if a RWE model might suggest a benefit or harm from statin therapy in patients with a variety of heart failure subtypes. The Intermountain study included 15,010 patients with heart failure (Table 1) who were treated with a variety of statin therapies and doses (Figure 1). While not explicitly stated, the data from this study appear to have evaluated those with systolic heart failure. The investigators examined overall differences across several end points between patients treated with statins vs those not treated (Figure 2). The team at Intermountain also evaluated the impact of statin therapy in primary and secondary prevention cohorts, exploring whether the presence of established ASCVD might impact outcomes. The end points chosen included: 1) the major adverse cardiovascular events (MACEs) of death, myocardial infarction, and stroke as a composite, as well as individual end points of: 2) MACE + heart failure hospitalization, 3) death, 4) myocardial infarction, 5) ischemic stroke, and 6) hospitalization for heart failure. Patients treated with statins had substantially fewer MACE events over the follow-up period: 52.1% vs 60.5%, ARR (absolute risk reduction, 8.4% (*P* < 0.0001) HR 0.53 (95% CI: 0.51-0.56)). Figure 2 illustrates the consistent and robust reductions associated with statin use across each of the end points. MACE + heart failure hospitalization was significantly lower: HR 0.41 (95% CI: 0.39-0.43), ARR 18.1%, *P* < 0.0001; death was significantly lower, HR 0.57 (95% CI: 0.51-0.62), ARR 6.2%, *P* < 0.0001; myocardial infarction was significantly lower, HR 0.41 (95% CI: 0.34-0.48), ARR 2.6%, *P* < 0.0001; ischemic stroke rates were lower, HR 0.54 (95% CI: 0.46-0.63), ARR 1.7% *P* < 0.0001 and rates of HF hospitalization were significantly lower, HR 0.41 (95% CI: 0.39-0.43), ARR 20.7%, *P* < 0.0001. Separate examinations of these end points in the secondary as well as primary prevention subgroup analysis were consistent with the overall data, demonstrating a robust reduction in clinical events. In summary, there were no subcategories of patients who did not benefit from statin therapy.

**Table 1:** Key Variables Across the Intermountain Study, the CORONA and GISSI-HF Trials

	Intermountain Study	CORONA Trial	GISSI-HF
Sample size	15,010	5,011	4,574
% ischemic heart failure	33%	100%	40%
Median age (y)	68	73	68
Female (%)	34%	23.5%	22.6%
Median follow-up (y)	4.5	2.73	3.9
Type 2 diabetes (%)	39.5%	29.5%	26%
History of percutaneous coronary intervention or coronary artery bypass grafting	Not reported	25.9%	21.6%
Prior myocardial infarction	21.2%	60%	32.8%
Prior stroke	5.9%	12.5%	4.5%

The Intermountain data are extremely impressive and provocative, and expand what we know about statin use in heart failure beyond the evidence of CORONA and GISSI-HF. There is likely some degree of bias that cannot be accounted for statistically despite multivariable modeling and best efforts by the investigators. Nonetheless, these data demonstrate a consistent reduction across cardiac, cerebrovascular, and heart failure-associated clinical events and lower overall mortality in those treated with statins. These data add a RWE perspective to a growing body of evidence supporting a benefit from statin use in heart failure patients.

## Why might these data be strongly supportive of statin use in patients with heart failure when 2 large, well-executed randomized clinical trials failed to show a benefit?

First, we should acknowledge that the CORONA trial did support a “benefit trend” in patients randomized to statin therapy, albeit only an 8% relative risk reduction. But, one should always consider the impact of external generalizability on clinical trial data. There is always a potential for randomized trials to fail to select the appropriate patient groups to study leading either to erroneous conclusions or an inability to generalize to populations not well represented in the trials under evaluation. RWE supplements data from randomized trials by clarifying efficacy questions when narrowly defined trials fail to answer such question and RWE broadens our understanding with regard to efficacy and safety.

Second, the Intermountain study had a longer duration of follow-up than either the CORONA or GISSI-HF trials. The median follow-up was 4.5 years, substantially longer than the 2.73 years in GISSI-HF and modestly longer than the 3.9 years in CORONA. A substantial number of the Intermountain patients were followed for an even longer period. It is important to realize that statins work by preventing ASCVD-related clinical events, yet their benefit is dependent on adequate exposure (lipid reductions and anti-inflammatory actions) over time. The recent trends, including with GISSI-HF and CORONA, to design outcomes trials with a shorter (2-3 years) time period to evaluate an investigational product vs placebo or comparator potentially biases studies toward placebo or comparator, not the active product under evaluation if potential benefit is not allowed time to adequately accrue. Many trials now utilize models that incorporate event-driven goals with a minimum or fixed amount of follow-up time which allows for robust safety and efficacy determinations.

Third, some have criticized the CORONA and GISSI-HF trials as having an overly selective or biased participant type of enrollment. The broadly captured population under study by Intermountain reinforces the generalizability of the findings and helps clinicians understand how statin therapy is useful in heart failure.

## What can we learn from the Intermountain study?

First, the data are generalizable to the broad population of patients we treat with systolic heart failure. This was not a subselected, narrowly focused population. The patients evaluated resemble those we evaluate and treat in clinic or at the hospital. In this vein, we applaud the work by the Intermountain group to cast a broad and inclusive net capturing statin use across a wide-range of participants who resemble the patients we treat daily in clinical practice. Second, it is not essential to use maximum doses of high intensity statins to see a clinical benefit. Third, data from the Intermountain study convincingly demonstrate that statin therapy in systolic heart failure patients is safe and well tolerated. Fourth, the evidence also strongly demonstrates an association of benefit in those on statin therapy across a range of cardiovascular end points. Finally, the data also remind us that statins are underutilized in this population, even within an excellent medical center system like Intermountain system.

## Should we change our practice?

Will clinicians see such dramatic risk reductions with statins in the heart failure population if we follow the data from Intermountain and prescribe statins more widely in this population? Perhaps or perhaps not. It is important to acknowledge that 2, well-designed randomized clinical trials failed to demonstrate convincingly any benefit from statins. At a minimum, the Intermountain study should stimulate each of us to pause and critically reflect on our own practices with regard to the use of statins in heart failure patients. The Intermountain data support the use of statins with regard to safety and efficacy. These data and the work by the team at Intermountain should stimulate discussion at the NIH with regard to funding a third clinical trial, adequately powered, pragmatically designed and implemented, to evaluate the potential benefit of statins in heart failure if skeptics of statin use remain. Furthermore, more research is needed with regard to measuring the impact of statin therapy in HF subtypes such as systolic and diastolic subgroups, populations previously excluded due to racial and/or other factors and to generate data with regard to longer term follow-up for duration of statin efficacy and importantly the impact of statin noncompliance on disease mitigation. Maybe the Intermountain group will address some of these concerns in a future study.

In summary, we commend Anderson et al and the team at Intermountain Health for a novel study design which practically readdressed this issue. Their design was robust using conservative evaluation techniques with multivariable modeling and implemented across a health system with a diverse patient population. Additionally, they did not overinterpret their findings. We congratulate them for bringing to our attention as a clinical community once again the importance of statin therapy in the heart failure population.

## Funding support and author disclosures

Dr Wright is an advisory board member for Novartis, Novo Nordisc, and Boehringer Ingelheim.
